# The Fog Horn Nuisance

**Published:** 1894-05

**Authors:** 


### THE FOG HORN NUISANCE.

WITH the opening of navigation the citizens of Buffalo are again tortured by the almost continual sounding of a ponderous fog horn located within the environs of our harbor. We have heretofore, in these columns, called attention to the serious menace to health that this infernal machine causes. We even go further and affirm, without reservation, that it is destructive to human life.

In these days of worn and tired nerves, a good night's sleep is needed to fit men and women for the work of tomorrow. We are now speaking of those persons who, judged by ordinary standards, are classified as enjoying good health. Yet, owing to the perplexities of business, with its many cares and anxieties, most of such are light sleepers and are easily awakened by undue and unusual noises. With the first toot of the fog horn they are aroused and sleep returns to them no more for the night. Hence, they reach their business offices next day with jaded nerves and tired muscles, and are wholly unequal to the duties before them.

But, if those in good health are thus disturbed, what shall we say for the sick? It requires no longdrawn argument to prove that these, in innumerable instances, are made infinitely worse by the unnecessary tootings of the fog horn, and, in some cases, we doubt not, they are driven to insanity or death.

It is utterly idle for navigators or vessel owners to contend that it is necessary to keep a populous city, numbering 300,000 inhabitants, in a state of perturbation that menaces health and life, in order to preserve the few vessels that may approach Buffalo harbor in a fog during the lake season. In most countries, vessels come to anchor when approaching a harbor during a fog, and here if a strong search light were properly erected on the site of the fog horn it would meet every requirement of safety to life and property.

We speak seriously. This is an important matter to the business interests of Buffalo, let alone the questions of life and health, to which we have before referred. Well-to-do people will leave Buffalo early in the Spring, and return late in the Fall, thus prolonging their outings for the purpose of avoiding this everlasting fog horn din, hence spend thousands of dollars in other places that would be used to the benefit of Buffalo. Still others, who reside here as a matter of pleasure or comfort, will flee from this terrible noise nuisance and seek residences in cities where fog horns are unknown.

This is a matter for the Health Department to consider deliberately and with great gravity. If the local authorities are not clothed with adequate power to deal with it, then the offices of the State Board of Health should be invoked, to the end that the nuisance may be effectively and permanently abated. The United States Government has no more right to establish and maintain a nuisance within our borders than has an individual, and it should be called to account therefor just as summarily, just as certainly.

